# A novel pre-clinical strategy to deliver antimicrobial doses of inhaled nitric oxide

**DOI:** 10.1371/journal.pone.0258368

**Published:** 2021-10-13

**Authors:** Vinicius S. Michaelsen, Rafaela V. P. Ribeiro, Edson Brambate, Aadil Ali, Aizhou Wang, Layla Pires, Mitsuaki Kawashima, Yu Zhang, Anajara Gazzalle, Shaf Keshavjee, Lorenzo Del Sorbo, Marcelo Cypel

**Affiliations:** Latner Thoracic Research Laboratories, Toronto General Hospital Research Institute, University Health Network, University of Toronto, Toronto, Ontario, Canada; Heidelberg University Hospital, GERMANY

## Abstract

Effective treatment of respiratory infections continues to be a major challenge. In high doses (≥160 ppm), inhaled Nitric Oxide (iNO) has been shown to act as a broad-spectrum antimicrobial agent, including its efficacy *in vitro* for coronavirus family. However, the safety of prolonged *in vivo* implementation of high-dose iNO therapy has not been studied. Herein we aim to explore the feasibility and safety of delivering continuous high-dose iNO over an extended period of time using an *in vivo* animal model. Yorkshire pigs were randomized to one of the following two groups: group 1, standard ventilation; and group 2, standard ventilation + continuous iNO 160 ppm + methylene blue (MB) as intravenous bolus, whenever required, to maintain metHb <6%. Both groups were ventilated continuously for 6 hours, then the animals were weaned from sedation, mechanical ventilation and followed for 3 days. During treatment, and on the third post-operative day, physiologic assessments were performed to monitor lung function and other significative markers were assessed for potential pulmonary or systemic injury. No significant change in lung function, or inflammatory markers were observed during the study period. Both gas exchange function, lung tissue cytokine analysis and histology were similar between treated and control animals. During treatment, levels of metHb were maintained <6% by administration of MB, and NO_2_ remained <5 ppm. Additionally, considering extrapulmonary effects, no significant changes were observed in biochemistry markers. Our findings showed that high-dose iNO delivered continuously over 6 hours with adjuvant MB is clinically feasible and safe. These findings support the development of investigations of continuous high-dose iNO treatment of respiratory tract infections, including SARS-CoV-2.

## Introduction

Nitric oxide (NO) is an endogenously produced molecular mediator, serving important roles, such as a blood vessel relaxant and a non-specific toxic defense molecule against infectious organisms at various sites, including the lungs [1,2]. Based on these properties, the therapeutic role of exogenous NO delivery has been studied in pre-clinical models [3,4], and clinical settings [5]. Currently, inhaled NO (iNO) at low doses (10–40 ppm) is routinely used to treat pulmonary hypertension, hypoxemia after lung transplantation, and acute respiratory distress syndrome (ARDS), in which continuous administration at these doses appears to be safe [6]. Recent investigations have further explored its use as an antimicrobial therapy [7]. When delivered in high concentrations, nitric oxide covalently binds to important cellular components of microorganisms such as RNA, DNA, proteins, and lipids, thereby inhibiting or killing target pathogens mostly through nitrosative stress [8], nitrosylation [1], or oxidative damages by attributed reactive byproducts [9]. Previous *in vitro* studies have identified the antimicrobial effects of NO to be optimal when given ≥160 ppm continuously for a period of approximately 6 hours. Under these conditions, complete elimination of all microorganisms has been reported [10–14]. However, potential safety issues concerning the delivery of continuous high-dose iNO limited its application to clinical settings. These concerns are related to 1) formation of metHb, 2) formation of toxic nitrogen dioxide (NO_2_), and 3) lung inflammation. In order to overcome this, some investigators have used high-dose iNO with 30–60 min intermittent administrations [15]. Similarly, high-dose iNO is being studied in several clinical trials for SARS-CoV-2 (NCT 04306393, 03331445, 04338828, 04305457, 04476992, and 04312243, ClinicalTrials.gov), all limited to 30 min intermittent administrations [16,17]. While being safe and potentially efficacious based on *in vitro* data [14], these delivery regimes tend to be less efficient when translated to clinical studies [7,15,18–20]. We have recently demonstrated that 12 hours continuous administration of 200 ppm of iNO during acellular normothermic *ex vivo* lung perfusion (EVLP) did not induce any alteration in lung function or inflammation. However, the *ex vivo* platform and the absence of blood in this system prevent us from studying the potential *in vivo* systemic toxicity of high-dose iNO treatment [21].

Thus, the present study explores the feasibility and safety of delivering *in vivo* continuous antimicrobial doses of iNO for extended periods in a pre-clinical large animal model.

## Material and methods

### *In vivo* animal guidelines and standards

Animal care and experimental protocol were approved by the Toronto General Hospital Research Institute Animal Care Committee. All animals received humane care in compliance with the Principles of Laboratory Animal Care formulated by the National Society for Medical Research and the Guidelines for the Care and Use of Laboratory Animals prepared by the Institute of Laboratory Animal Resources [22].

### Experimental procedures and study design (Fig 1)

**Fig 1:**
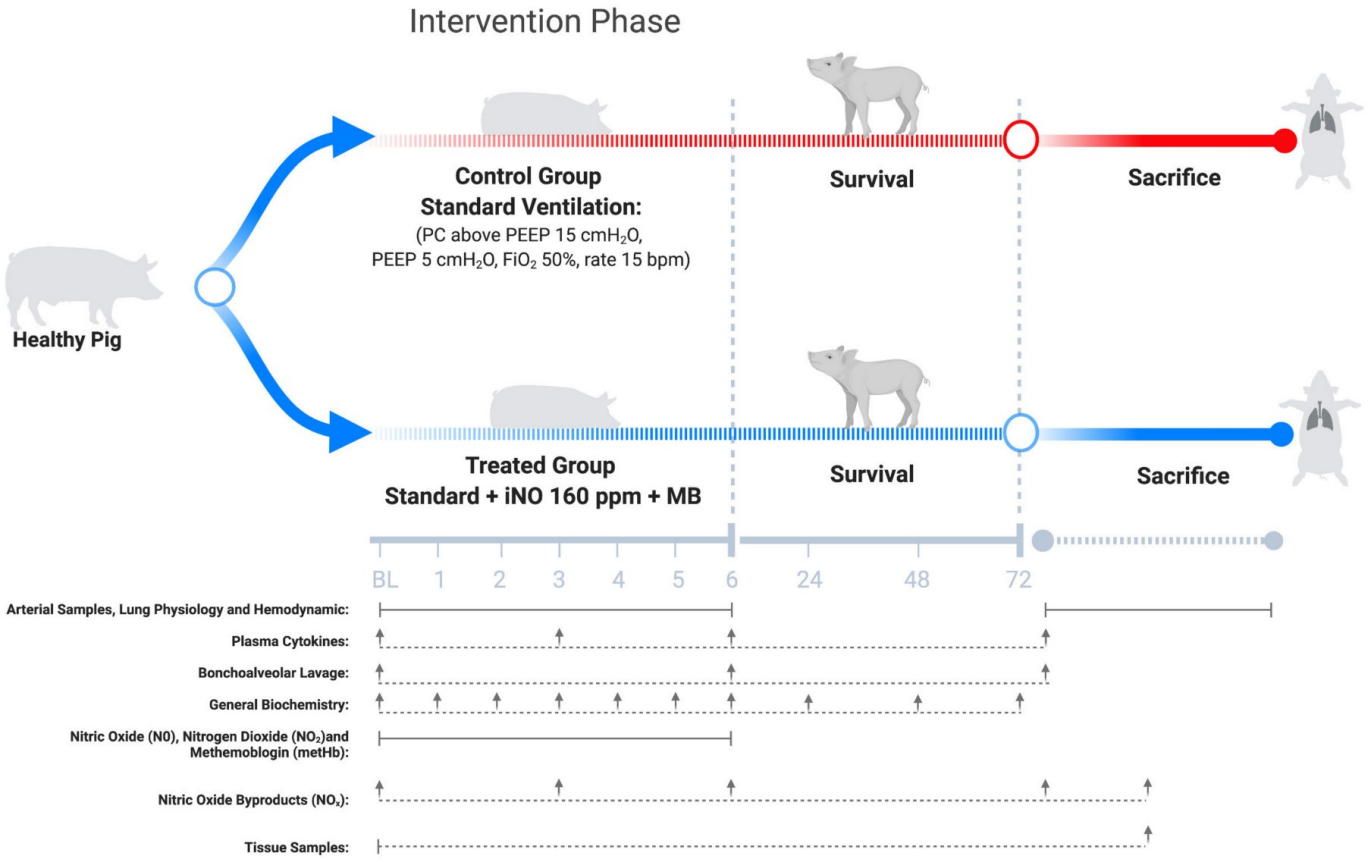
Study design. Ten animals were randomly allocated into one of the following groups: Control group (red line, n = 5), which consisted of 6h of standard ventilation; or treatment group (blue line, n = 5), which consisted of 6h of standard ventilation + 160 ppm of iNO + MB 1mg/Kg administered in bolus when metHb was 6% or higher. After the intervention phase, all animals were weaned, extubated and followed clinically for three days. On day 3, animals were sacrificed by exsanguination. Samples collection and assessments were performed during the experiments detailed in the lower part of the Fig 1. Definition of abbreviations: BL = baseline, ppm = parts per million, MB = methylene blue, iNO = inhaled nitric oxide, metHb = methemoglobin, NO_2_ = nitrogen dioxide and NOx = nitric oxide byproducts, PC = pressure control, PEEP = positive end-expiratory pressure, bpm = beats per minute.

Ten male 10-week old Yorkshire x Duroc/Landrace pigs were sedated, and a peripheral ear venous line was placed. Under general anesthesia with continuous IV infusion of propofol, the animals were intubated and connected to an intensive care unit (ICU) ventilator (Maquet Servo-I, Solna, Sweden). Ventilation settings were the following: positive end-expiratory pressure (PEEP) = 5 cmH_2_O, pressure control above PEEP = 15 cmH_2_O, respiratory rate = 15 bpm, FiO_2_ = 50%, considered as standard ventilation in this study. Invasive arterial and central venous lines were placed and used to monitor blood pressure and central venous pressure, respectively. The airway of all animals was inspected using a fiberoptic bronchoscope, followed by bronchoalveolar lavage (BAL) of the right lower (RLL) lobe using 30 mL of normal saline. Saline solution was given IV at a rate of 10 mL/kg/h to maintain homeostasis and central venous pressure (CVP) within a physiological range (4 to 6 mmHg). Intravenous cefazolin was administered at a single dose of 1g at the beginning of the procedure.

After lines were in place and BAL was collected, the animals were turned into a prone position, and after 5 minutes, baseline parameters were recorded, including ventilatory parameters, arterial blood gases, metHb levels. Plasma was also collected. Animals were then randomly allocated into one of the following two groups: (1) control group (n = 5), which consisted of 6 hours of standard ventilation; (2) iNO group (n = 5), which consisted of 6h of standard ventilation + 160 ppm of iNO + 1 mg/kg methylene blue (MB) (02230770, Omega Laboratories, MTL, Canada), whenever required, to maintain metHb <6%. A heated humidifier system (MR850JHU, MR290HFV, Fisher and Paykel Healthcare, AKL, New Zealand) was added to both ventilation strategies during the experiment. At the end of the 6 hours, pigs were turned back to supine position, and post-intervention BAL was collected from a similar location to that of the baseline sample. During the recovery phase, ventilatory support was managed in a staged fashion from pressure control to pressure support. The endotracheal tube (ET) was kept in place until deep reflexes (e.g. cough) were observed. Once extubated, the animals were transferred to our animal care housing facility and closely monitored until they could stand up, walk, and eat independently. All animals were followed up for three days. On the third postoperative day, animals were sedated with propofol and remifentanil infusions, and intubated (7.5mm COVIDEN) by performing a tracheostomy through a midline cervical incision, as described previously by our group [23]. Bronchoscopy was performed, and a BAL was obtained from the RLL. To gain access to the thoracic cavity, a sterno-laparotomy was performed, both pleural cavities were opened, and lungs were macroscopically evaluated. After 5 minutes of ventilation with FiO_2_ = 100%, arterial blood samples were obtained directly from the aorta. Biopsies were taken from the RLL after sacrificing the animal by exsanguination under deep anesthesia.

### Sample size

Ten male 10-week old Yorkshire x Duroc/Landrace pigs were divided into two groups of five each, as shown previously. The reasoning for the small sample size was that continuous high-dose iNO was evaluated *in vivo* for the first time in our study. Our objective was to gather basic evidence using this therapeutic gas in a pre-clinical experimental design. In addition, cost and logistics to perform large animals were taken into account, and the decision was to reserve our sample size to the minimal possible number while demonstrating the concept of our approach. This n = 5 is typically acceptable on experiments of this nature.

### Assessment of pulmonary function

Assessments of lung compliance and arterial blood gas analysis were performed every 30 minutes during the 6 hours of mechanical ventilation and postoperative day three.

### Delivery and monitoring of nitric oxide and nitrogen dioxide(Fig 2)

**Fig 2:**
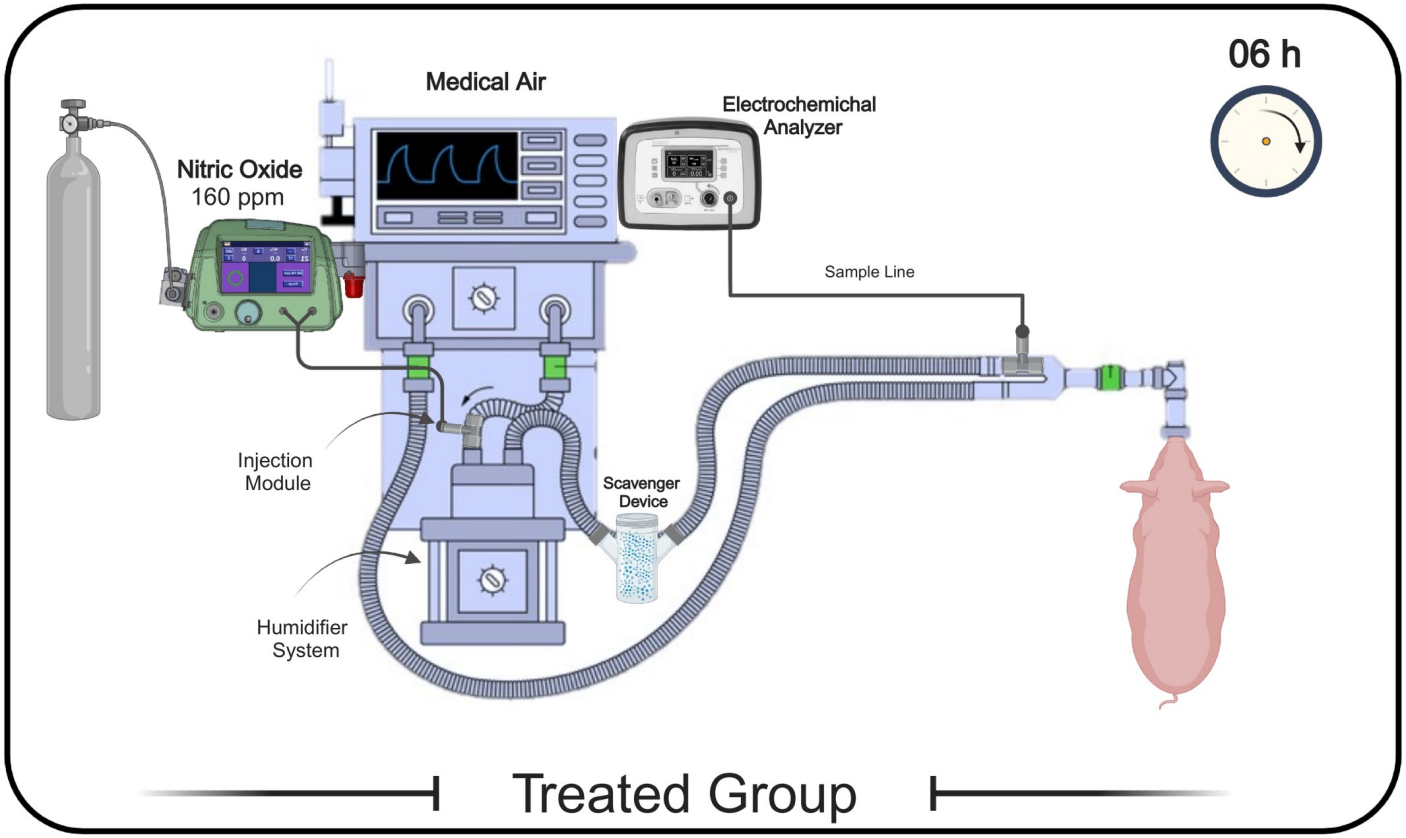
Schematic representation of the inhaled nitric oxide delivery system, and its components location. iNO was delivered for 6 hours continuously to reach the target dose of 160 ppm using a medical-grade pressurized cylinder of 2,000 ppm with a custom digital gas regulator device. The gas was administered through a connector attached to the breathing circuit to deliver NO during predetermined ventilation settings using an ICU ventilator. Inspiratory NO and NO_2_ levels were monitored in real-time using a dedicated gas electrochemical analyzer sampling from a port of the inspiratory limb of the ventilatory circuit, proximal to the endotracheal tube. A multi-absorber scavenger device was used to maintain NO_2_ below a safety threshold (<5 ppm), and was placed right after the humidification system. Definition of abbreviations: ppm = parts per million, iNO = inhaled nitric oxide, NO_2_ = nitrogen dioxide, ICU = intensive care unit, h = hours.

In group 2, iNO was delivered for 6 hours continuously to reach the target dose of 160 ppm using a medical-grade pressurized cylinder of 2,000 ppm (Praxair, Inc. ON, Canada) with a custom digital gas regulator device (12^th^ Man Technologies Inc., CA, United States). The gas was administered through a connector attached to the breathing circuit to deliver NO during predetermined ventilation settings using an ICU ventilator (Maquet Servo-I, Solna, Sweden). Inspiratory NO and NO_2_ levels were monitored in real-time using a dedicated gas electrochemical analyzer (AeroNOx, International Biomedical, TX, United States) sampling from a port of the inspiratory limb of the ventilatory circuit, proximal to the endotracheal tube, separated from the exhaled gas to avoid measuring exhaled NO_2_. A multi-absorber scavenger device (M1173310, Medisorb, Vyaire, HEL, Finland) was used to maintain NO_2_ below a safety threshold (<5 ppm), and was placed right after the gas injector module, and of the humidifcation system. The AeroNOx analyzer was calibrated before each experiment based on the manufacturer’s guidelines and specifications. The parameters were recorded every 30 minutes and continuously monitored during the entire NO treatment.

### Measurement of methemoglobin and total nitrite/nitrate (NO_x_)

MetHb was measured at baseline, every 30 minutes during the procedure and on the third postoperative day (sacrifice), from arterial blood samples using a blood gas machine (Rapidpoint 500, Siemens Healthcare, Canada), and expressed as a percentage of total hemoglobin. Nitrite and nitrate, also known as (NO_x_), were measured in plasma (baseline, 3, 6, and 72h), BAL (baseline, 6, and 72h), and lung tissue (72h). NO_x_ was determined using the modified Griess method as described by Tatsch E. *et al*. [24] in combination with Griess Reagent System Protocol (G2930, Promega, WI, United States). The visible absorbance of NO_x_ derivatives was measured at 530 nm by a spectrometer (SpectraMax Plus, Molecular Devices, CA, United States). Values were expressed as μM of total nitrite and nitrate in plasma and BAL samples. Tissue values were normalized to total protein measured with Pierce BCA protein assay (23225, Thermo Fisher Scientific, IL, United States) and expressed as μM/mg of tissue.

### Sample collection protocol

BAL was collected from the RLL at baseline, 6, and 72 hours. Plasma samples were taken at baseline, 3, 6, and 72 hours, snap-frozen, and stored at -80°C for subsequent analyses. Blood samples were taken hourly during the experimental procedure and on each day of the follow-up period (24, 48 and 72 hours) for blood biochemical analyses consisting of creatinine, urea, alanine aminotransferase (ALT), aspartate aminotransferase (AST), lactate dehydrogenase (LD), prothrombin time (PT), international normalized ratio (INR), activated partial thromboplastin time (aPTT) were measured at the Laboratory Medicine Program, University Health Network. Lung tissue samples from the right lower lobe were divided to be either snap-frozen and stored at -80°C or formalin-fixed and paraffin-embedded for histological analysis. Blood gases, including pH, glucose, and lactate, were measured every 30 minutes during the ventilation period using a blood gas machine. Lung function, hemodynamic parameters (heart rate (HR), mean systemic arterial pressure (MAP)), oxygen saturation (SpO_2_), and partial pressure of arterial oxygen/fraction of inspired oxygen ratio (PaO_2_/FiO_2_) were also recorded every 30 minutes. Hemodynamic parameters were monitored using (CARESCAPE Monitor B650, GE Healthcare, Helsinke, FI).

### Inflammatory cytokine assays

Pro-inflammatory cytokines were measured in plasma, tissue, and BAL samples. Lung tissue samples were homogenized according to previously described methods [25]. Tissue lysates and plasma were assayed using ELISA kits for the following porcine inflammatory interleukins (ILs) markers: IL-1β (PLB00B, R&D Systems, MN, United States), IL-6 (P6000B, R&D Systems, R&D Systems, MN, United States), and IL-8 (P8000, R&D Systems, R&D Systems, MN, United States). The assay was performed according to the manufacturer’s instructions, and values were expressed in pg/mL. Tissue cytokine concentrations were normalized to total protein and expressed as pg/mg of tissue.

### Histologic examination and lung injury scoring

Lung tissue was fixed with 10% phosphate-buffered formalin and embedded in paraffin. They were sectioned and stained using the standard hematoxylin and eosin staining method. We compared the two groups with respect to interstitial edema, intra-alveolar edema, as well as parenchymal and airway integrity of the structure. Briefly, a score scaled from 0 to 3 represents the severity of lung injury: 0 for no or very minor; 1 for mild and limited; 2 for intermediate; and 3 for widespread or prominent injury. The analysis was performed by one of the study authors in a blinded manner (MK).

### Statistical analysis

All results are expressed as mean ± standard deviation, and each data plotted in the graphs. For longitudinal data, a mixed-effects model was performed for analysis between groups. Mann-Whitney U test was performed to compare differences between groups regarding lung injury score and cytokines at each time point. A *p-value* less than *0*.*05* was considered to be significant. Graph Pad Prism Version 8 (GraphPad Software, La Jolla, CA) was used to conduct all statistical analyses.

## Role of funding source

The Toronto General Hospital Foundation supported this work. The funding source had no involvement in study design, collection, analysis, and interpretation of data, writing of the report, and the decision to submit the paper for publication. All authors confirm that they had full access to all the data in the study and accept responsibility during the submission.

## Results

### Nitric oxide delivery and nitrogen dioxide formation

**Fig 3:**
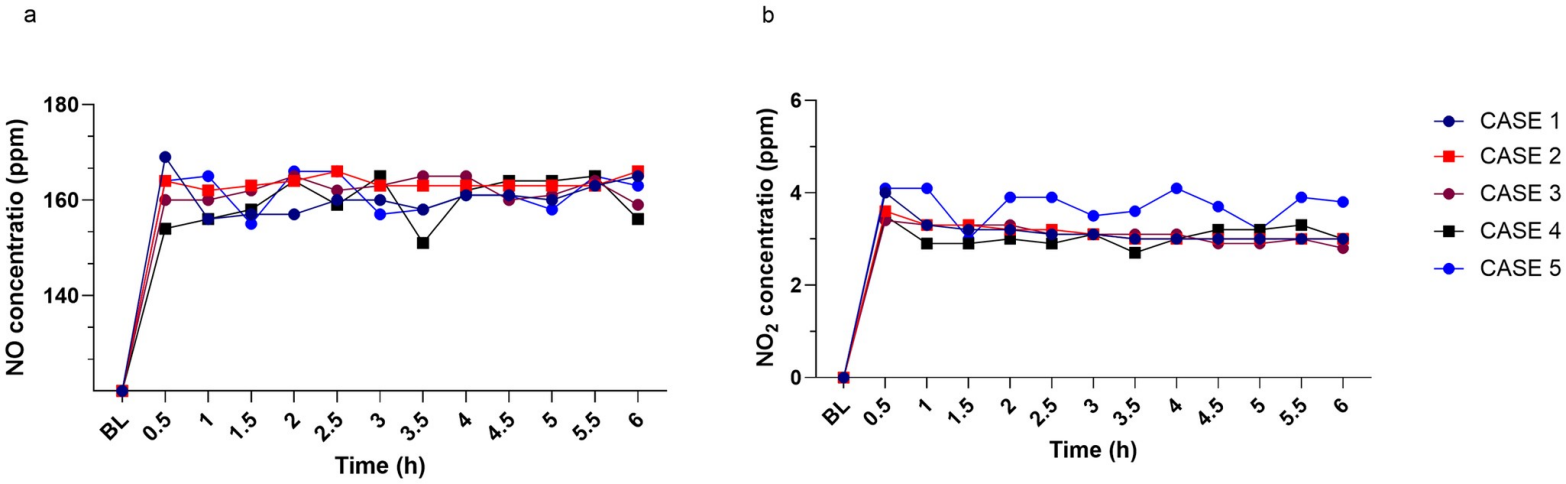
High-dose nitric oxide (NO) delivery and nitrogen dioxide (NO_2_) formation. Various parameters of the delivery system were monitored during 6 h of continuous exposure to a pre-set concentration of NO (160 ppm). (a) The NO concentration in the inspiratory line monitored with an electrochemical analyzer (AeroNOx, International Biomedical, TX, United States). The maximum variation observed was 161.6 ± 3.53 ppm, with the highest dose reaching 169 ppm of the pre-set concentration. (b) The buildup of nitrogen dioxide was monitored during the same period of exposure. The NO2 level remained below the safety threshold (5.0 ppm), during the entire experiment, with an average level of 3.24 ± 0.35 ppm. Data are presented as single data points from each individual case: CASE 1 (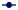), CASE 2 (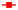), CASE 3 (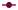), CASE 4 (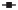), and CASE 5 (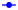). Definition of abbreviations: BL = baseline, ppm = parts per million, NO = nitric oxide, and NO_2_ = nitrogen dioxide.

The average delivered iNO was 161.6 ± 3.53 ppm, with the highest dose reaching 169 ppm (Fig 3a). Levels of NO_2_ remained below the safety threshold (5 ppm) during the entire experiment, with an average level of 3.24 ± 0.35 ppm (Fig 3b). This indicates that the NO_2_ scavenger added to the delivery system was efficient and did not compromise iNO administration.

### Formation of metHb and NO byproducts (NO_x_)

**Fig 4:**
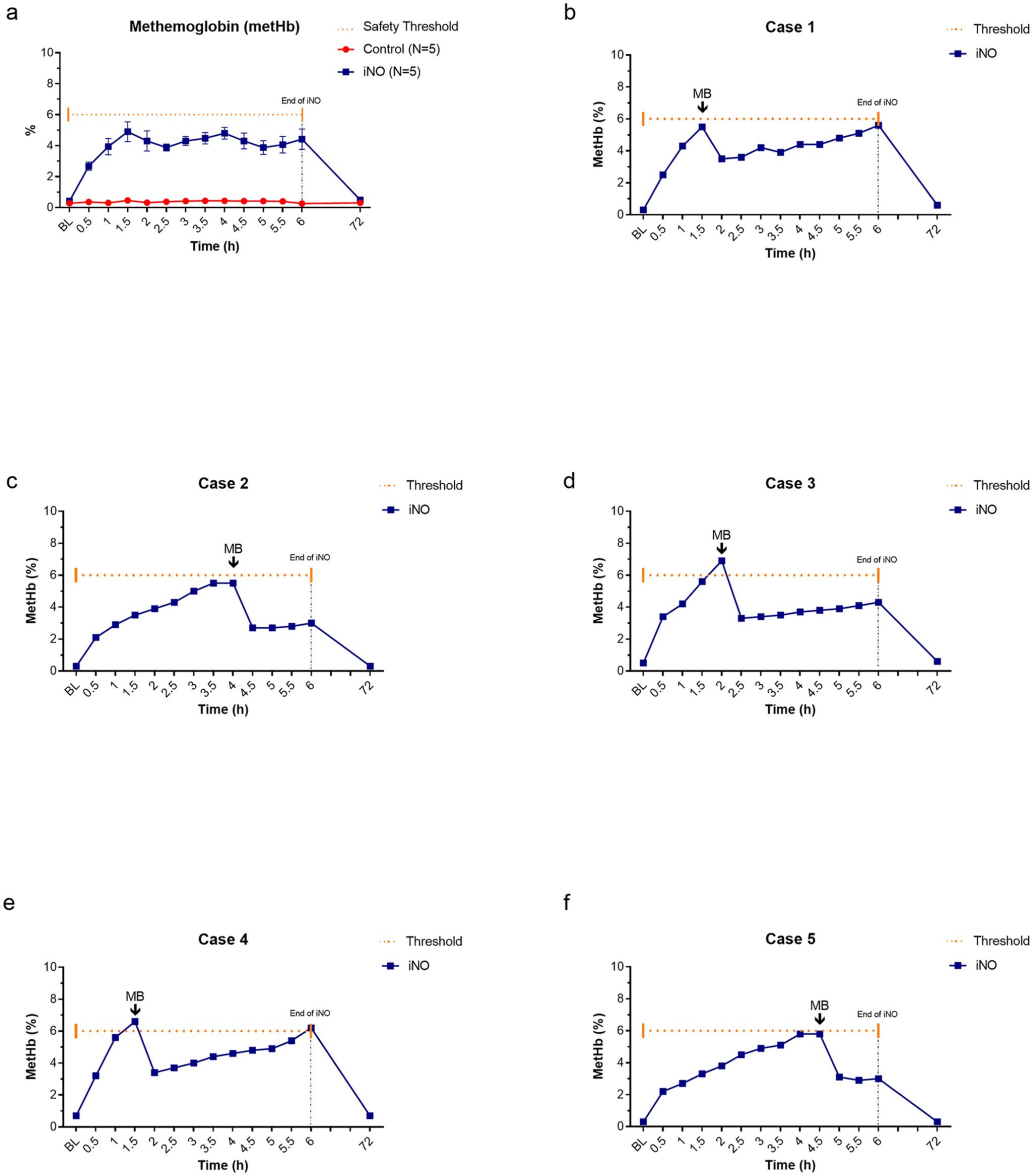
Methemoglobin (metHb) levels over time. MetHb was measured from an arterial blood sample every 30min during the intervention phase. Inhaled nitric oxide (iNO)-treated animals had significantly higher levels of metHb than controls (a). Variation over time of the metHb percentage and time of methylene blue administration is shown for each treated animal (b-f). The safety threshold for metHb was set at 6%. One dose of methylene blue (MB) during the entire intervention was sufficient to keep metHb levels below the safety threshold (6%). Definition of abbreviation: BL = baseline, MB = methylene blue, metHb = methemoglobin, iNO = inhaled nitric oxide, and ns = non-significant.

During the administration of iNO, we observed increased levels of metHb (maximum level 6.9%), which were rapidly decreased with MB treatment (Fig 4a). The average time from iNO initiation to the administration of MB was 162 min. One dose of MB was sufficient to maintain metHb levels below 6% in each animal of the iNO group. Fig 4(b)–4(f) shows the levels of metHb formation over time and the respective timing of MB administration for each treated case.

**Fig 5:**
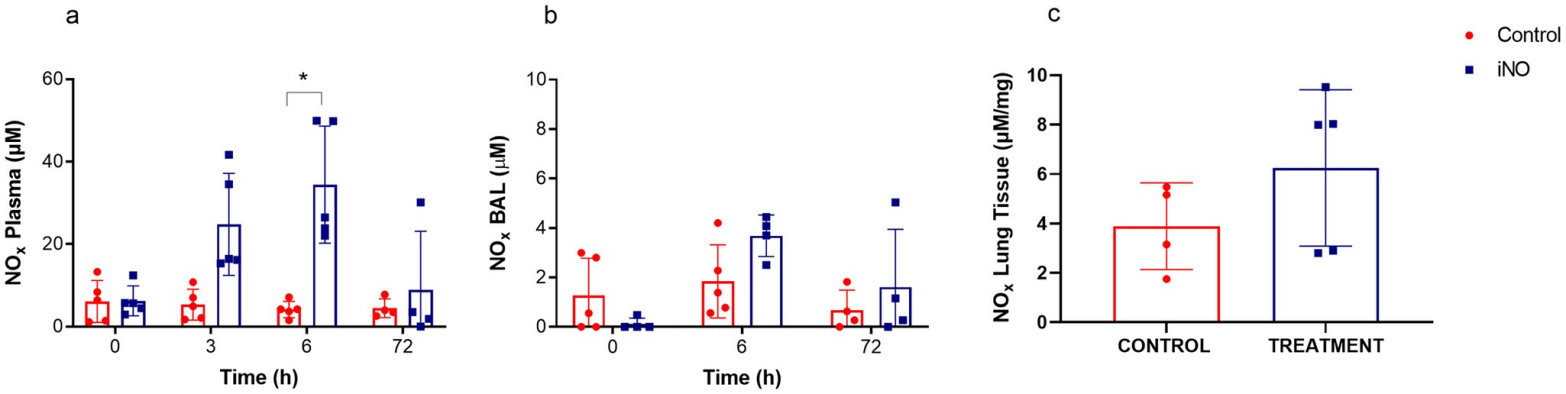
Nitric oxide byproducts formation (NO_x_) overtime in control versus iNO treated animals. The uptake of iNO was estimated by monitoring NO_x_ concentrations in plasma, BAL and lung tissue. NO_x_ levels were measured using modified Griess method in combination with Griess reagent system. (a) NO_x_ gradually increased in plasma over time and was significantly higher in the iNO group (blue, n = 5) at 6h when compared to control (red, n = 5) *p = 0*.*032*, consistent with iNO delivery. After 72 hours, levels were back to normal and comparable to controls. We did not notice any significant differences in NO_x_ levels in BAL (b) and lung tissue (c). Graphs represented through interleaved scatter with bars with individual values plotted as mean with standard error of mean. * = statistically significant. Definition of abbreviation: BAL = bronchoalveolar lavage, iNO = inhaled nitric oxide, and NO_x_ = nitric oxide byproducts.

To evaluate NO uptake, we measured the circulatory stable byproducts of NO, nitrite, and nitrate (NO_x_). NO_x_ increased during the course of iNO delivery in plasma and BAL and returned to baseline levels three days after the intervention, which is consistent with efficient iNO delivery. Tissue levels of NO_x_, although not statistically significant, tended to be higher in the treatment compared with the control group (Fig 5).

### Pulmonary function, inflammation, and histologic examination

**Fig 6:**
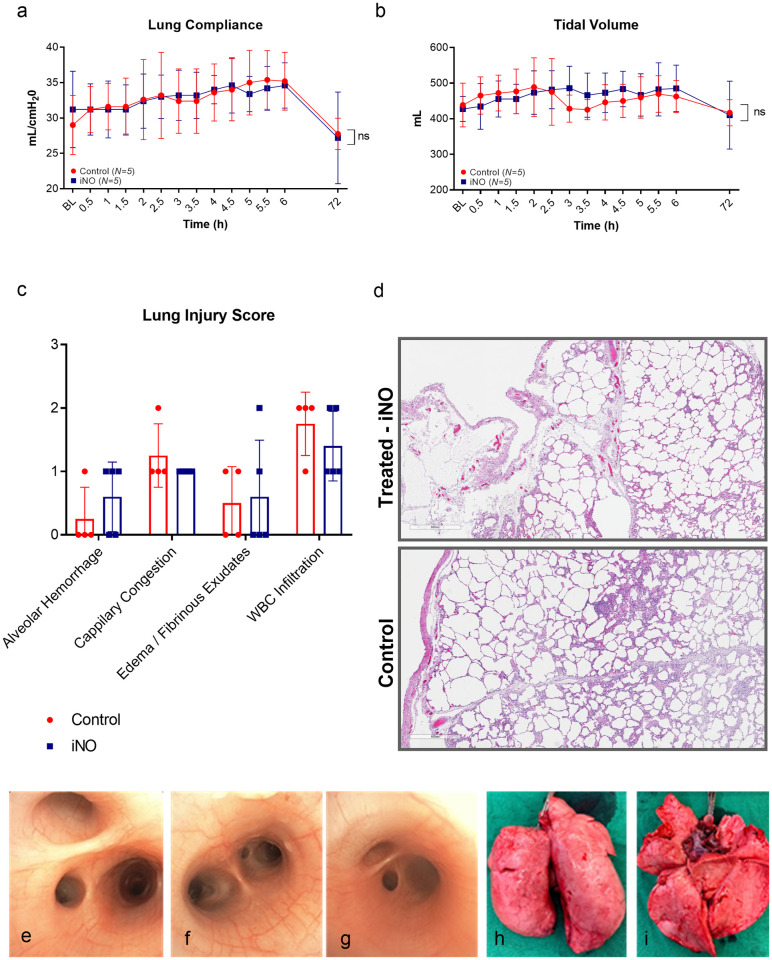
Pulmonary function, inflammation analysis, and histologic examination of the control group (red box/line, n = 5) versus treatment iNO group lungs (blue box/line, n = 5). The following lung functional parameters were evaluated: (a) lung compliance, (b) tidal volume. There was no significant difference between control and iNO treated animals. Acute lung injury score (c) was similar between the two groups in each evaluated component: Alveolar hemorrhage, capillary congestion, edema and WBC infiltration. Representative images (5X) of H&E stained biopsies of one control (bottom image) and one iNO treated animal (upper image) taken at 72 hours are shown in (d). Additionally, representative bronchoscopic images of one iNO-treated case at baseline (e), after 6 hours of iNO (f), and after three days of survival (g) show no signs of airway injury. Furthermore, after sacrifice, lungs were explanted for visual inspection, and gross appearance is shown in (h-i). No acute lung injuries were noted. Data displayed as mean ± SD for each time-point. Definition of abbreviation: BL = baseline, iNO = inhaled nitric oxide, WBC = white blood cells, H&E = hematoxylin & eosin, and ns = non-significant.

The pulmonary function did not reveal any acute deterioration or abnormality during the delivery phase of iNO (Fig 6a–6i). Histologic examination and acute lung injury scoring were evaluated, as described previously [26]. None of the lung samples showed any evidence of pulmonary edema, inflammatory cell infiltration or other signs of lung injury (Fig 6d). Acute lung injury scores were similar in the two groups regarding alveolar hemorrhage *(p =* 0.823); capillary congestion *(p = 0*.*862)*; edema/fibrinous exudates *(p = 0*.*999)*; white blood cells (WBC) infiltration *(p = 0*.*823)*. Representative pictures of the bronchoscopic assessment and lung gross appearance of the iNO group are shown in (Fig 6e–6i), which shows no evidence of airway injury, corroborating the histological findings (Fig 6d). Accordingly, overall levels of the pro-inflammatory cytokines IL-1β (*p = 0*.*961*), IL-8 *(p = 0*.*346)*, and IL-6 *(p = 0*.*070)* in plasma, IL-1β (*p = 0*.*604*), IL-8 *(p = 0*.*808)*, and IL-6 *(p = 0*.*986)* in BAL, and IL-1β (*p >0*.*099*), IL-8 *p (>0*.*099)*, and IL-6 *(p = 0*.*563)* in lung tissue, demonstrated no differences between the two groups. Plasma IL-6 concentrations at 6 hours were significantly higher in control compared to the iNO group (1631 ± 124 pg/mL vs 580±214, *p = 0*.*01*), but at 72 hours were similar in the two groups (control 717 ± 209 pg/mL vs iNO 547 ± 244 pg/mL) (Table 1).

**Table 1:** Cytokine levels in plasma, bronchoalveolar lavage and lung tissue at baseline, 3, 6, and 72 hours.

Plasma Cytokine Levels	Control (pg/mL)	Treatment (pg/mL)	*P* value
**BL**			
**IL-1β**	534±202	228±123	*0*.*547*
**IL-6**	851±331	589±279	*0*.*309*
**IL-8**	0	0	*>0*.*999*
**3**^**rd**^ **hour**			
**IL-1β**	483±72	500±188	*>0*.*999*
**IL-6**	581±121	296±163	*0*.*150*
**Il-8**	0	0	*>0*.*999*
**6**^**th**^ **hour**			
**IL-1β**	120.5±23	161±77	*0*.*690*
**IL-6**	1631±124	580±214	*0.*01*
**IL-8**	0	16±16	*>0*.*999*
**72 hours**			
**IL-1β**	164±67	185±67	*>0*.*999*
**IL-6**	717±209	547±244	*0*.*746*
**IL-8**	0	4±4	*>0*.*999*
**BAL Cytokines Levels**			
**BL**			
**IL-1β**	96±58	48±23	*0*.*976*
**IL-6**	0	0	*>0*.*999*
**IL-8**	31±20	109±84	*0*.*682*
**6**^**th**^ **hour**			
**IL-1β**	1895±634	2531±920	*0*.*730*
**IL-6**	120±77	137±53	*0*.*730*
**IL-8**	3166±730	3083±919	*0*.*730*
**72 hours**			
**IL-1β**	336±238	474±198	*0*.*555*
**IL-6**	41±41	24±10	*0*.*444*
**IL-8**	3166±730	3083±919	*0*.*730*
**Cytokine Levels in Tissue**			
**IL-1β**	103±66	96±42	*>0*.*999*
**IL-6**	3.4±3	15±10	*0*.*563*
**IL-8**	0	0	*>0*.*999*

Definition of abbreviations: **BL** = baseline, **IL-1β =** Interleukin 1 beta, **IL-6 =** Interleukin 6, **IL-8 =** Interleukin 8, **BAL** = bronchoalveolar lavage, **ns** = non-significant. Values are in mean ± standard deviation.

*Significant difference (*p < 0*.*05*).

### Hemodynamic parameters, arterial blood gas, and clinical conditions

Table 2 shows hemodynamic parameters and arterial blood gas analysis in the two groups. Overall, physiologic parameters (SpO_2_, PaO_2_/FiO_2_, PaCO_2_, pH, glucose, lactate) assessed during the experiment showed no differences between groups. Similarly, pH, glucose, and lactate remained stable within normal levels, without significant differences at 72 hours.

**Table 2:** Hemodynamic parameters and arterial blood gas analysis at baseline, 3, 6, and 72 hours.

	Control			Treatment			*P value*
	*BL*	*6h*	*72h*	*BL*	*6h*	*72h*	
***HR (bpm)***	102.6 ± 11.3	108.6 ± 16.7	85.8 ± 11.9	100.6 ± 10	124.4 ± 22.2	105.2 ± 12	*ns*
***MAP (mmHg)***	109.3 ± 15.3	104.3 ± 7.2	NA	91.7 ± 11.9	98.13 ± 18.9	NA	*ns*
**SpO**_**2**_ ***(mmHg)***	99.6 ± 0.5	99.6 ± 0.5	99.8 ± 0.5	98.8 ± 1.8	98.2 ± 1.8	98.2 ± 2.5	*ns*
**PaO**_**2**_**/FiO**_**2**_ ***(mmHg)***	488.2 ± 42.2	555.2 ± 15	543.5 ± 15.1	446.6 ± 37.3	511.4 ± 30.3	558.7 ± 45.6	*ns*
***PaCO***_***2***_ ***(mmHg)***	41.80 ± 6.4	31.1 ± 7	30.6 ± 6.7	41.9 ± 8.6	30.6 ± 2.1	27.8 ± 4	*ns*
***pH***	7.4 ± 0	7.5 ± 0.1	7.5 ± 0.1	7.4 ± 0.1	7.5 ± 0	7.5 ± 0.1	*ns*
***Glu (mmol/L)***	5.5 ± 1	7.6 ± 2.5	9.0 ± 2.5	5.6 ± 0.6	7.8 ± 2	8.3 ± 1	*ns*
***Lac (mmol/L)***	1 ± 0.6	1.8 ± 1.7	1.9 ± 0.8	0.9 ± 0.2	2.5 ± 2.3	2.2 ± 1.1	*ns*

Definition of abbreviations: **BL** = baseline, **HR** = heart rate, **MAP** = mean systemic arterial pressure, **SpO**_**2**_ = oxygen saturation, **PaO**_**2**_**/FiO**_**2**_ = partial pressure of arterial oxygen/ fraction of inspired oxygen, **PaCO**_**2**_ = partial pressure of carbon dioxide, **pH** = potential of hydrogen, **Glu** = glucose, **Lac** = lactate, **ns** = non-significant. Values are in mean ± standard deviation.

After 6 hours of exposure to high-dose iNO, we carefully observed the animals to check for any signs of possible rebound effects (e.g. hemodynamic instability, or hypoxemia). All pigs in both groups remained stable after weaning of mechanical ventilation. During the 3-day follow-up period, animals from both groups were clinically well. The pigs showed regular activity, appetite, and urinary output. One animal from the control group died unexpectedly during the recovery phase due to unknown reasons.

### Biochemical analyses

**Fig 7:**
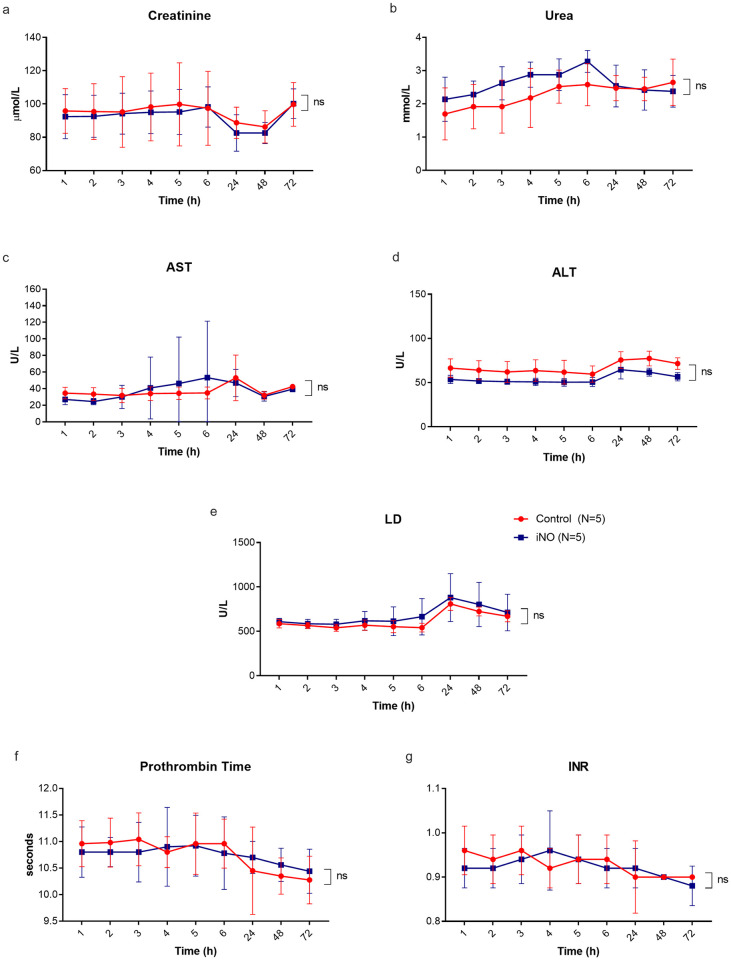
Biochemical analyses of control (red line, n = 5) versus iNO treated animals (blue line, n = 5). Venous blood samples were collected hourly during the intervention phase and daily during the follow-up period in animals of both groups. Kidney function markers, liver enzymes, and coagulation parameters were stable and similar in both groups. Data are displayed as mean ± SD for each time-point. Definition of abbreviation: AST = aspartate aminotransferase, ALT = alanine aminotransferase, LD = lactate dehydrogenase, PT = prothrombin time, INR = international normalized ratio, and ns = non-significant.

Kidney function markers, liver enzymes, and coagulation parameters were stable in both control and iNO treated animals and did not show significant difference at any time point during the study period (Fig 7). This indicates that the iNO treatment did not demonstrate any acute adverse effects or systemic toxicity on the animals.

## Discussion

This study demonstrates that the administration of continuous high doses of inhaled NO is feasible and safe. In a recent experimental study using an *ex vivo* lung platform, we already demonstrated that prolonged high-dose iNO did not cause any direct pulmonary injury [21]. However, due to the *ex vivo* nature of the study and the absence of blood perfusion in the system, the safety of high-dose iNO was limited to its potential direct effect on the lung parenchyma [23]. In the present study, we demonstrated the safety of continuous high-dose iNO delivery *in vivo*, highlighting the clinical relevance of the findings and the foreseeable clinical translation of this innovative therapeutic approach, including its potential application in the treatment of acute viral illness such as SARS-CoV-2. We primarily focused on the potential toxicity induced by iNO through the formation of metHb and NO_2_. We showed that an IV bolus of MB during high-dose iNO treatment was very efficient to prevent metHb formation to toxic levels above 6% without compromising the iNO dosing or causing any other adverse effects. Importantly, prior studies have shown that MetHb reductase in swine may have respond differently from human MetHb reductase, and a variation from human to human in MetHb reductase also can exist. In that regard, its recommended appropriate management, and continuous monitoring of MetHb using a dedicated non-invasive device (i.e SpMet, Masimo). Despite not having taken a measurement in real time in the present study, the q.30 min provided enough safety as demonstrated by absence of any secondary signs of tissue hypoxia such as high lactate.[27,28] Furthermore, although this study explored for safe alternatives involving continuous high-dose iNO administrations, an alternative to MB administration would be short pauses of iNO once MB increases above safe thresholds.

We also carefully monitored NO_2_ formation throughout the administration of iNO due to its known toxicity [29]. The amount of NO_2_ formed during inhalation of NO depends not only on the flow, fraction of inspired oxygen, and iNO concentration, but also on the contact time between them. As a safety measure, a scavenger device was added to the delivery system to keep inspiratory NO_2_ levels below 5 ppm, which proved to be very efficient in maintaining NO_2_ levels within target, even with a fraction of inspired oxygen 50%. It is important to note, that the Centers for Disease Control and Prevention (CDC) has recommended occupational limits for nitrogen dioxide in lower limits [30] however, our decision for NO_2_ levels threshold used in this experimental study, was based, on the most recent existing reference used for humans, including multicenter clinical trials. While our study demonstrated that limiting NO_2_ at 5ppm is safe for 6 hours, we cannot affirm that would be the case in longer or repeated administrations was needed.

Safety concerns regarding iNO have been previously reported. Studies have shown that iNO therapy may increase the risk of renal dysfunction, especially with prolonged use in patients with ARDS [31,32]. Furthermore, investigators have reported hypothetical systemic effects of inhaled NO on coagulation [33]. We closely monitored renal and liver functions, as well as coagulation parameters of all animals during the entire study, and we showed that these markers were clinically stable and similar between iNO-treated and controls animals. Furthermore, evaluation of lung function, inflammation, and histology showed no evidence of injury. Finally, no significant inflammatory response was induced by iNO treatment compared to controls, as indicated by low levels in plasma, BAL and lung tissue of key pro-inflammatory cytokines.

There is consistent *in vitro* evidence demonstrating that NO when delivered continuously at high doses (≥160 ppm) or using NO donors, can inhibit, or kill a broad list of microorganisms, including multi-drug resistant bacteria, and viruses, in a relatively short time [10,11,34]. However, the *in vivo* translation of these studies has been limited by the potentially deleterious effects of metHb and NO_2_ formation induced *in vivo* by iNO. To avoid these complications, several clinical protocols investigated the efficacy of high-dose iNO delivered *intermittently* for short periods rather than continuously. However while safe, few minutes only of delivery have compromised the antimicrobial full potential of the treatement [7,15,18–20,34–38]. The findings of our study demonstrated the safety of the *in vivo* continuous administration of high doses of iNO for 6 hours, supporting the potential clinical application of iNO delivery at effective antimicrobial doses as an innovative treatment strategy.

iNO has been reported as a promising therapeutic strategy for lung bacterial and viral infections [1,9] and a potential candidate to treat coronavirus infections, including COVID-19 [39]. Some studies have shown that NO inhibits the membrane fusion of offspring virus S protein binding to the host cell angiotensin-converting enzyme 2 (ACE2) receptor [40]. Interestingly, the same group reported that NO has a similar mechanism for SARS-CoV-2 [41]. Based on these findings, and considering the emergency imposed by the lack of treatment options, off-label applications have been explored, and high-dose iNO is being studied in several clinical trials for SARS-CoV-2 (NCT 04306393, 03331445, 04338828, 04305457, 04476992, and 04312243, ClinicalTrials.gov), all limited to 30 min intermittent administrations [16,17]. Along those lines and based on our current study, we initiated a clinical trial evaluating exactly the protocol described here in humans with severe SARS-CoV-2 (NCT04383002, ClinicalTrials.gov).

Our study has some limitations. First, we decided to investigate the effect of continuous high-dose iNO administration in a non-injured model (healthy animals) to better understand the isolated effect of the iNO treatment and elucidate possible injury, inflammation, and side effects. However, we acknowledge that the interaction between high iNO doses and lung inflammation demands further investigation. While we propose high-dose iNO as a potential antimicrobial therapy, we did not study its effect in an infection model at this moment, as we focused on its safety and feasibility. Our lab started exploring the efficacy of our strategy in *in vivo* large animal models of respiratory infection.

In conclusion, our findings show that high-dose (160 ppm) iNO delivered continuously over 6 hours combined with one dose of MB (1 mg/kg) is feasible and safe. These findings open new perspectives in the treatment of respiratory tract infections, including SARS-CoV-2.

## Supporting information

S1 Data Plos one data 2020. (XLSX)
